# Dense city centers support less evolutionary unique bird communities than sparser urban areas

**DOI:** 10.1016/j.isci.2024.108945

**Published:** 2024-01-18

**Authors:** Federico Morelli, Jiri Reif, Mario Díaz, Piotr Tryjanowski, Juan Diego Ibáñez-Álamo, Jukka Suhonen, Jukka Jokimäki, Marja-Liisa Kaisanlahti-Jokimäki, Anders Pape Møller, Leszek Jerzak, Raphaël Bussière, Marko Mägi, Theodoros Kominos, Antonia Galanaki, Nikos Bukas, Gábor Markó, Fabio Pruscini, Olaf Ciebiera, Yanina Benedetti

**Affiliations:** 1 Czech University of Life Sciences Prague, Faculty of Environmental Sciences, Kamýcká 129, CZ-165 00 Prague 6, Czech Republic; 2 Institute of Biological Sciences, University of Zielona Góra, Prof. Szafrana St. 1, PL 65-16 Zielona Góra, Poland; 3 Institute for Environmental Studies, Faculty of Science, Charles University in Prague, Staré Město, Czech Republic; 4 Department of Zoology and Laboratory of Ornithology, Faculty of Science, Palacky University in Olomouc, Olomouc, Czech Republic; 5 Department of Biogeography and Global Change, Museo Nacional de Ciencias Naturales (BGC-MNCN-CSIC), E-28006 Madrid, Spain; 6 Department of Zoology, Poznań University of Life Sciences, Wojska Polskiego 71C, PL-60-625 Poznań, Poland; 7 Department of Zoology, Faculty of Sciences, University of Granada, Granada, Spain; 8 Department of Biology, University of Turku, Turku, Finland; 9 Nature Inventory and EIA-services, Arctic Centre, University of Lapland, P. O. Box 122, FI-96101 Rovaniemi, Finland; 10 Ecologie Systématique Evolution, Université Paris-Sud, CNRS, AgroParisTech, Université Paris-Saclay, F-91405 Orsay Cedex, France; 11 Rue des Roses, 87200 Chaillac-sur-Vienne, France; 12 Department of Zoology, Institute of Ecology and Earth Sciences, University of Tartu, Tartu, Estonia; 13 Estonian Environmental Board, Roheline 64, 80010 Pärnu, Estonia; 14 Department of Zoology, School of Biology, Aristotle University of Thessaloniki, 54124 Thessaloniki, Greece; 15 Plegadis, Riga Feraiou 6A, 45444 Ioannina, Greece; 16 Department of Plant Pathology, Institute of Plant Protection, Hungarian University of Agriculture and Life Sciences, Ménesi út 44, 1118 Budapest, Hungary; 17 S. C. della Pantiera 23, 61029 Pantiera, Urbino (PU), Italy

**Keywords:** Ecology, Ornithology, Evolutionary biology

## Abstract

Urbanization alters avian communities, generally lowering the number of species and contemporaneously increasing their functional relatedness, leading to biotic homogenization. Urbanization can also negatively affect the phylogenetic diversity of species assemblages, potentially decreasing their evolutionary distinctiveness. We compare species assemblages in a gradient of building density in seventeen European cities to test whether the evolutionary distinctiveness of communities is shaped by the degree of urbanization. We found a significant decline in the evolutionary uniqueness of avian communities in highly dense urban areas, compared to low and medium-dense areas. Overall, communities from dense city centers supported one million years of evolutionary history less than communities from low-dense urban areas. Such evolutionary homogenization was due to a filtering process of the most evolutionarily unique birds. Metrics related to evolutionary uniqueness have to play a role when assessing the effects of urbanization and can be used to identify local conservation priorities.

## Highlights

• Urban bird assemblages in seventeen European cities were surveyed
• We compared species assemblages in a gradient of building density (low, medium, and high)
• We used the average evolutionary distinctiveness (ED) score to compare assemblages
• Dense city centers supported birds’ ED scores lower than in low-dense urban areas

## Graphical abstract

**Figure:**
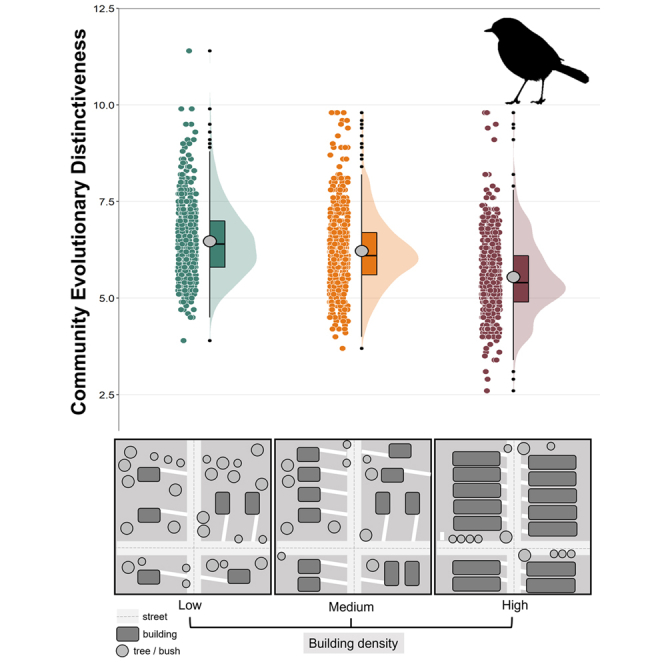

## Introduction

The expansion and growth of urban areas consume the land, increase the human footprint, and transform drastically the landscapes.^1^^,^^2^ Specifically, urban is considered one of the fastest-growing land-use types globally.^3^ This trend is expected to continue globally, with potential negative effects on biodiversity.^4^ Birds have a long history of being used as a target group for studies in urban ecology.^5^^,^^6^^,^^7^^,^^8^ Earlier comparisons between avian communities from urban and more natural environments nearby generally indicate a decline in the total number of species.^9^^,^^10^^,^^11^ This is mainly because urbanization increases habitat fragmentation and reduces the amount of available habitat for several wildlife species,^12^^,^^13^ although an increasing number of species are being favored by urbanization in recent times.^14^ However, the species richness approach used to assess the effect of urbanization on overall biodiversity is limited by its failure to take into account the ecological roles of species and the different contributions they make to ecological communities.^15^ For example, even assuming a loss in the overall number of species, cities can attract other urbanphilic or urban tolerant species.^16^^,^^17^^,^^18^ This exchange of species can determine intrinsic changes in avian communities, leading to their biotic homogenization, which is the replacement of specialist species, often by more generalist species in terms of their functional traits.^19^^,^^20^ Briefly, cities offer habitats with characteristics that favor the occurrence of synurbic species (e.g., species which have greater urban than rural population densities).^21^ Additionally, previous studies also suggested a significant decrease in the evolutionary uniqueness or distinctiveness of bird assemblages in urban areas compared to rural or natural environments.^22^^,^^23^^,^^24^ Evolutionary uniqueness is a species-specific property, a measure that indicates the evolutionary isolation of a given species.^25^^,^^26^ Briefly, an evolutionarily distinct species (e.g., characterized by a relatively high ED score) has an evolutionary history that is shared by few other species in the complete phylogenetic tree of birds (including all bird species from the world).^27^ Such a measure was suggested as a useful tool for prioritizing evolutionary history in conservation planning.^28^^,^^29^

By configuration and structure, cities can be heterogeneous environments for wildlife. Typically, the amount and spatial arrangement of urban green spaces are related to the distribution of biodiversity within the cities.^30^ The biodiversity can be linked to the ecosystem’s potential resilience^31^^,^^32^ and the human well-being of residents.^33^^,^^34^ Biodiversity conservation is strongly linked to the availability of data on wildlife and the correct assessment of the species diversity. Considering the rapid expansion of urban areas, mitigating the expected loss in biodiversity depends on a correct understanding of how urbanization affects the biological communities and the subsequent development of wildlife management strategies that incorporate urban ecosystems.^35^^,^^36^

Assessing the evolutionary distinctiveness of bird communities in a gradient of urbanization within cities can provide important information for the conservation of urban birds.^7^ In rural areas, bird species from recently diversifying clades appeared to be better “exploiters” of such habitats, being virtually more urban-tolerant species.^37^ Highly unique species, on the other hand, represent an essential target for conservation, since their loss cannot be easily compensated within a species assemblage,^38^ and because species-specific biotic interactions may also be unique, at least regionally. We expect changes in the number of evolutionary unique species and overall uniqueness of bird assemblages in different city areas because more unique species are associated with specific ecological characteristics. A previous European large spatial-scale study comparing bird species uniqueness between urban and rural areas^24^ already showed the potential influence of different urban features on the evolutionary uniqueness of bird communities. Some species, characterized by a relatively high evolutionary uniqueness (e.g., European robin *Erithacus rubecula* and Eurasian hoopoe *Upupa epops*), were found to be linked to the presence of large urban trees or bushes.^24^^,^^39^^,^^40^ Then, the configuration of the different urban areas of the city can shape the avian composition in terms of evolutionary uniqueness.^7^ This information is important for correctly developing strategies to protect more unique bird species. Thus, when possible, it could help mitigate the overall decline of urban biodiversity, indicating where conservation efforts should be focused. Birds are among the organisms most vastly affected by urban expansion.^17^^,^^20^^,^^36^

In the present study, we investigate if the intensity of urbanization (i.e., degree of building density) is associated with bird communities showing lower levels of evolutionary distinctiveness. To do so, we collected a large dataset of urban birds in several European cities and compared their evolutionary distinctiveness in a gradient of urban density. In addition, we identified the top five bird species most typical of each type of urban area and compared their evolutionary heritage among urban types.

### Methods

#### Study area and data collection

**Figure 1:**
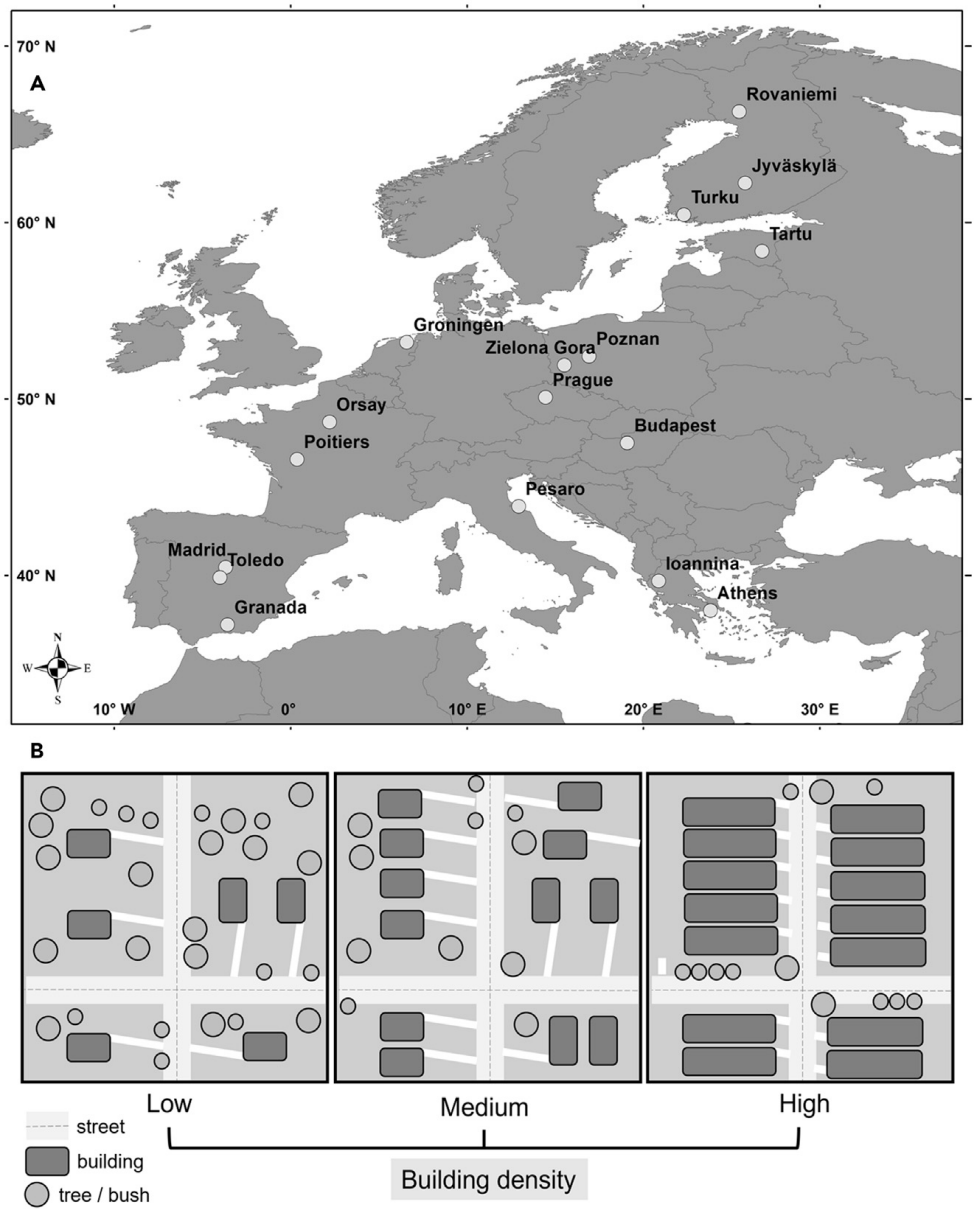
Study area and urbanization gradient The seventeen European cities focused on this study (A) and the classification of the urban areas in terms of building density and arrangement (e.g., level of urbanization) (B).

Seventeen cities located along a continent-wide latitudinal gradient, encompassing ten European countries from the Mediterranean area up to the Artic Circle, were surveyed in this study (Figure 1A). We considered urbanized areas as those with a percentage of built-up surface higher than 50%, as suggested by Marzluff et al..^41^ Within each city, three types of urban areas were identified based on the spatial arrangement and the density of building elements, and the coverage of green areas. Urban density can be defined as the concentration of building infrastructure within a certain urban area.^42^ Observers classified all surveyed urban areas using the following three categories (henceforth called “urban level”): Low (e.g., few isolated buildings such as family houses, less dense arrangement, with medium/large green areas), medium (e.g., more buildings with blocks of flats, closer each other, with small green areas or gardens) and high (e.g., very dense building areas, almost without the presence of green spaces, mostly represented only by trees on the roadside, for example, city centers) (Figure 1B). This classification is a revisit of the scheme presented in Urban Wildlife Management (Fig. 3.4),^43^ and is followed in a previous European study on urban ecology.^44^ Figure S1 is a graphical exploration of the differences in the overall cover of buildings and urban greenery in each type of area within the city, classified following its urban level (low, medium and high). This classification presents some advantages if compared to a more quantitative measure, such as the total percentage of built-up surface. Our classification combines the amount and spatial arrangement of buildings and green elements, typical of urban areas, providing a more accurate description.

In each city, a single ornithologist collected data on bird species composition through standardized point counts^45^^,^^46^ during the 2018 breeding season. Point counts were locally adjusted to correspond to the peak of the breeding season based on the local experts’ knowledge (e.g., May in southern Spain or June in Finland) to reduce bird detectability issues.^47^ Point counts were evenly distributed in the three types of urban levels in each city (low, medium and high), separated by a minimum of 150 m to avoid double-counting bird species. A total of one hundred 5-min point counts were visited in each city, only under good weather conditions (e.g., sunny day, weak or absent wind). Only expert ornithologists performed the fieldwork to reduce bird detection issues potentially associated with observers’ skills.^48^ All species seen or heard within a 50 m fixed radius circle were recorded, except for nocturnal species which were excluded from the analysis.

For each point count (e.g., bird community), with the matrix of bird occurrence, we calculated the total number of bird species (species richness)^49^ the community evolutionary distinctiveness (CED) or average evolutionary distinctiveness,^50^ and the maximum ED score. The CED is based on the evolutionary distinctiveness (ED) score, which is a measure of evolutionary history or uniqueness regarding the level of isolation of a species on the phylogenetic tree.^27^^,^^51^ The ED score is calculated as the total phylogenetic diversity of a clade, divided among its members.^27^^,^^51^ Each ED score unit is a million years.^51^ Here, we used ED scores extracted for all bird species of the world at www.edgeofexistence.org and based on the bird phylogeny published in Jetz et al..^27^ Using the ED score for each bird species present in a community, we estimated the CED as the average ED, considering all species belonging to the community. The CED of a community is calculated as follows:

$$\text{CED}=\sum_{i=1}^{n}\frac{{ED}_{i}}{N_{tot}}\text{,}$$

Where ED_*i*_ is the evolutionary distinctiveness score for each species *i*, divided by the number of all species recorded in the community, *N*_*tot*_.^24^^,^^50^

### Statistical analyses

To investigate the changes in CED of bird assemblages at each level of urbanization, we ran a generalized linear mixed effects regression model.^52^ The response variable (CED) was log-transformed to meet a normal distribution^53^ (Figure S2). The model predictors were: bird species richness (because of the potential correlation between CED and the total number of species), latitude, and level of urbanization (low, medium, high), while “city” was included as a random effect in the model to account for possible differences among cities and alleviate any potential spatial autocorrelation. The model was fitted using the package “lme4” for R,^54^ while the model output was obtained using the package “jtools” for R.^55^ Potential multicollinearity among predictors was assessed using the variance inflation factor (VIF)^56^ in the “car” package for R,^57^ on a generalized linear version of the model (e.g., excluding random factors). Only predictors with VIF<1.5 were incorporated into the model procedure^58^ (Table S2). The goodness of fit of the model was assessed by employing the conditional and marginal coefficient of determination for generalized mixed-effect models. Both measures were calculated using the function “r.squaredGLMM” from the “MuMIn” package for R.^59^

All statistical tests were performed using R software version 4.1.1.^60^

## Results

Within the 17 European cities surveyed, we found a total of 134 bird species (Table S1). Overall, the most common urban birds were house sparrows *Passer domesticus* (present in 59% of the total point counts), blackbirds *Turdus merula* (43%), common swifts *Apus apus* (42%), great tits *Parus major* (38%), wood pigeons *Columba palumbus* (34%), and feral pigeons *Columba livia* (34%) (Table S1). The top five birds, mostly characteristic of each type of urban area based on the building density in European cities, are shown in Table 1. Within the low-density urban areas, there are species with comparatively high ED scores, such as European robins *Erithacus rubecula* (ED score = 9.847) and blackcaps *Sylvia atricapilla* (ED score = 7.883) (Table 1). Within the medium-dense urban areas also were found species characterized by relatively high ED scores as Eurasian chaffinchs *Fringilla coelebs* (ED score = 9.945) and Western house martins *Delichon urbicum* (ED score = 7.955) (Table 1). The highest values of ED scores for birds in highly dense urban areas were for common swifts *Apus apus* and black redstarts *Phoenicurus ochruros* (ED scores = 6.725 and 5.304, respectively) (Table 1).

**Table 1:** Top-five bird species most frequently observed at each level of urbanization (low, medium, and high) in European cities, common name and evolutionary distinctiveness score (ED)

Species	Name	ED^27^	Urban level
*Phoenicurus phoenicurus*	Common redstart	5.560445	low
*Erithacus rubecula*	European robin	9.846652	low
*Passer montanus*	Tree sparrow	5.324101	low
*Sylvia atricapilla*	Blackcap	7.882945	low
*Turdus pilaris*	Fieldfare	4.800242	low
*Fringilla coelebs*	Eurasian chaffinch	9.946202	medium
*Delichon urbicum*	Western house martin	7.95514	medium
*Serinus serinus*	European serin	4.701469	medium
*Streptopelia decaocto*	Eurasian collared dove	4.991769	medium
*Turdus merula*	Blackbird	6.109976	medium
*Columba livia*	Feral pigeon	4.510291	high
*Corvus monedula*	Western jackdaw	3.86124	high
*Apus apus*	Common swift	6.724703	high
*Passer domesticus*	House sparrow	4.033652	high
*Phoenicurus ochruros*	Black redstart	5.304206	high

**Figure 2:**
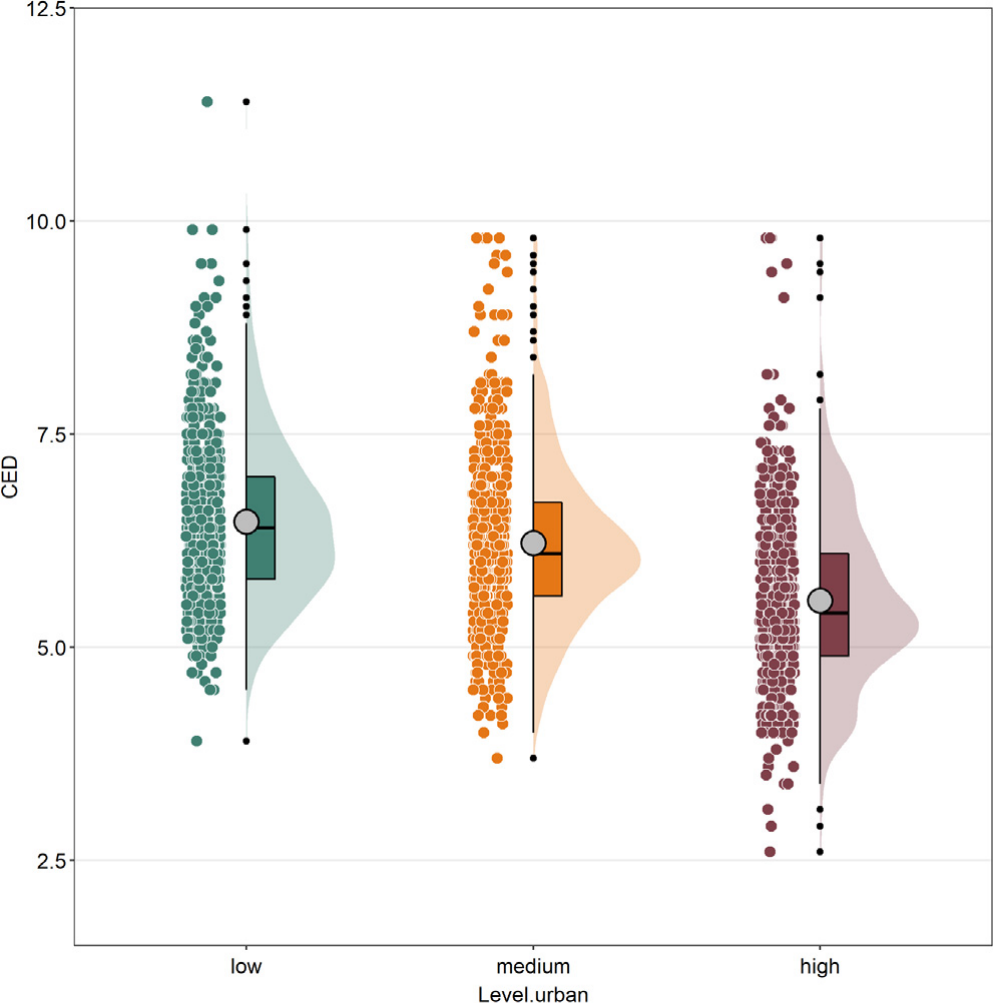
Mean values of evolutionary distinctiveness scores in avian communities (community evolutionary distinctiveness or CED) in a gradient of urbanization (low, medium and high) within seventeen European cities The raincloud plot shows the raw data, probability density and summary statistics such as the median (black bar in the middle of the colored rectangles), mean (gray circles), upper and lower quartiles by presenting individual data, a violin plot and a boxplot together.

**Figure 3:**
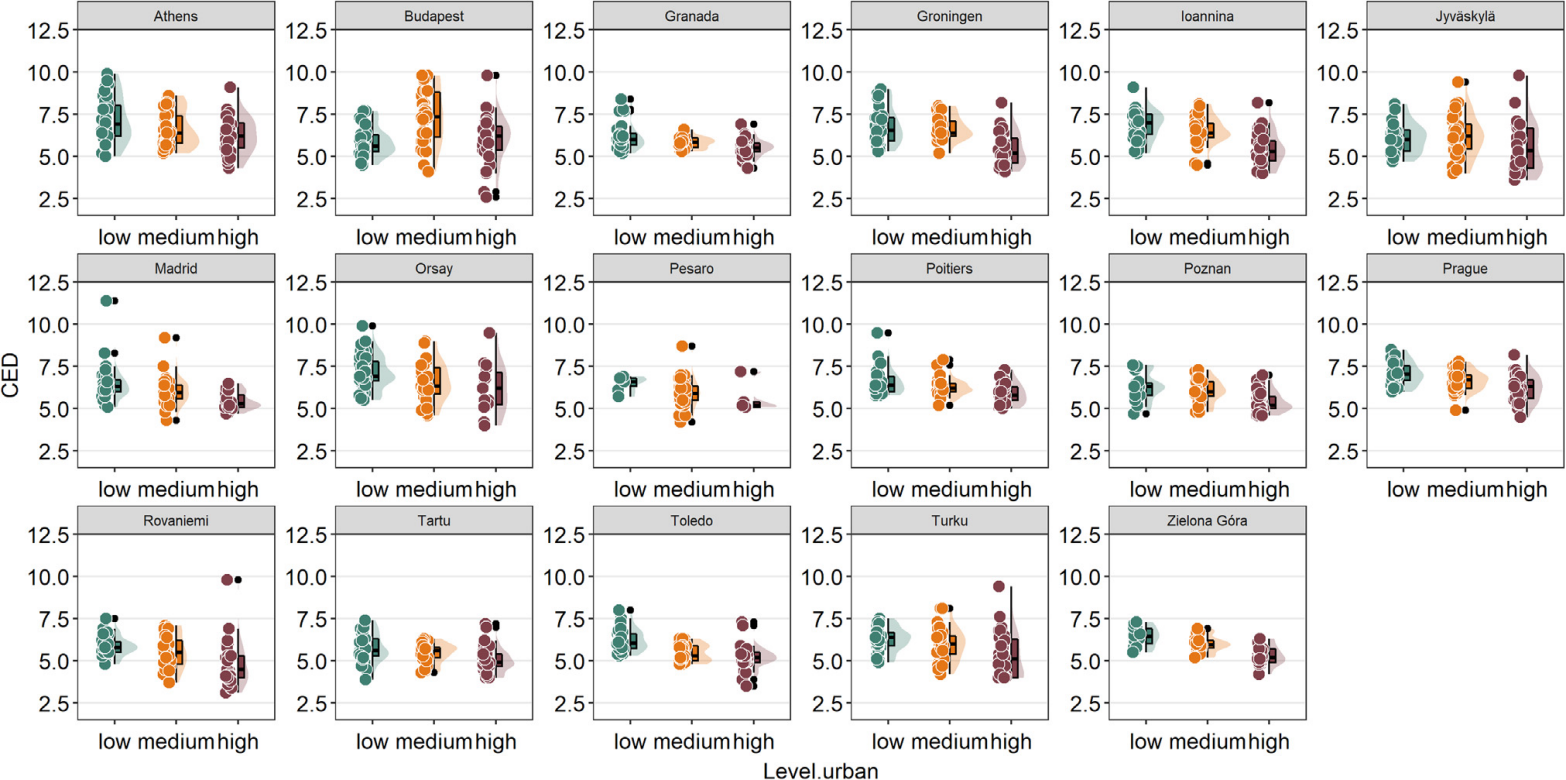
Mean values of evolutionary distinctiveness scores in avian communities (community evolutionary distinctiveness or CED) in a gradient of urbanization (low, medium, and high) in each European city The raincloud plot shows the raw data, probability density and summary statistics such as the median (black bar in the middle of the colored rectangles), upper and lower quartiles by presenting individual data, a violin plot and a boxplot together.

Bird communities living in high-dense urban areas were characterized by a lower evolutionary distinctiveness than those from medium and low-density urban areas (Figure 2), with around one million years less evolutionary uniqueness than communities from low-dense urban areas (Figure 2). When plotting the maximum ED scores for bird communities within the cities, we found a similar pattern than for CED (e.g., lower ED max in high dense urban areas than in medium or low dense urban areas) (Figure S3). The same pattern (e.g., lower CED in high-dense urban areas than in medium or low-dense urban areas) was almost constant in all studied European cities (Figure 3).

The results of the generalized linear mixed model showed a slightly positive but significant association between CED and the total number of species in the assemblages. Additionally, the results confirmed a significant and negative effect of high and medium-dense urban areas in CED (Table 2), even considering the effect of species richness. At the same time, the latitude of European cities was not correlated with changes in CED (Table 2).

**Table 2:** Results of a generalized linear mixed model assessing the community evolutionary distinctiveness of bird assemblages in European cities concerning the total number of bird species (richness), the latitude and the level of urbanization (low, medium and high)

Variable	Estimate	S.E.	t value	d.f.	p value
(Intercept)	1.785	0.025	70.761	26.67	<0.001
**Species richness**	**0.008**	**0.002**	**4.686**	**1120.64**	**<0.001**
**Level urban (medium)**	**−0.024**	**0.011**	**−2.143**	**1236.47**	**0.032**
**Level urban (high)**	**−0.122**	**0.013**	**−9.517**	**1238.59**	**<0.001**
Latitude	0.000	0.000	0.885	10.86	0.395

The city was included as a random factor to control for possible consistent differences among European cities. Significant variables are highlighted in bold. S.E. = standard error, t value = values of the statistic, d.f. = degree of freedom and p value. The model Pseudo-R^2^ = 0.283.

## Discussion

Bird species can respond differentially to the main effects of the urbanization process, depending on their ecological requirements^61^ and dispersal abilities.^62^^,^^63^ As a consequence, we can assume community changes related to the characteristics of urban areas.^10^^,^^13^^,^^64^ Conservation initiatives have already targeted the recognition and mitigation of reducing the functional diversity of avian communities in urban areas.^65^ Only a few studies focused on the evolutionary homogenization of avian communities but mainly comparing avian communities from urban with rural or natural habitats.^22^^,^^23^^,^^24^ Here, on the contrary, we compared the degree of bird’s evolutionary homogenization in different areas of the cities. The conservation of the evolutionary heritage of species is an important tool for establishing new conservation priorities.^51^ For this reason, nature conservation more often involves the protection of the phylogenetic diversity of bird communities.^27^^,^^66^ Currently, it is also recognized that protecting the avian evolutionary history can help to maintain ecosystem functioning and, consequently, human well-being.^67^

We found that highly densely developed areas of European cities (e.g., city centers) sustain bird communities with drastically reduced evolutionary uniqueness. Overall, urban bird assemblages from more dense building areas supported one million years less evolutionary history than communities from low-dense urban areas. Such evolutionary homogenization is mainly due to the filtering of bird species with the highest evolutionary distinctiveness scores. Among the species most characteristic from the highly dense urban areas of the cities, we found birds with ED scores rather low (e.g., species more phylogenetically closely related to other bird species) (Table S1). When exploring the values of ED score for all bird species recorded in this study, most of the species typical from high-dense city centers (e.g., occurrence in high-dense urban areas above 50%), are characterized by relatively lower ED scores than birds which are more frequent in low-dense urban areas. This effect is independent of the total number of species in the bird’s communities. Now, the protection of the evolutionary history in bird assemblages could be beneficial in terms of increasing the probability of sampling trait diversity among species and probably providing some specific human-centric benefits.^68^ However, the level of evolutionary uniqueness could also be related to the potential resilience of the whole assemblage, considering that evolutionarily distinct species are more prone to go extinct than less evolutionarily distinct species.^37^ In agricultural systems, for example, the occurrence and abundance of evolutionarily distinct bird species were associated with the presence of residual forests^24^ or diversified agricultural systems.^37^ In urban parks, some birds with comparatively high ED scores were linked to the abundance of large and old trees.^39^ Within the European cities, the three bird species with the highest ED scores were Eurasian hoopoes *Upupa epops* (ED score = 26.7), common firecrests *Regulus ignicapilla* (ED score = 22.8) and common little bitterns *Ixobrychus minutus* (ED score = 20.6). All those species were found mainly or exclusively in urban areas characterized by low building density and high amounts of different types of green layers (Table S1).

The presence of some bird species within the cities, rather unique in terms of evolutionary distinctiveness, could improve the community’s capacities to absorb rapid land use changes, also facing eventual climate change scenarios.^69^ Consequently, we claim that vegetation (e.g., urban forests, large trees along roadsides, etc.) and lower building density areas within European cities play an essential role in maintaining diversity across the tree of life. Specifically, urban forests and other green areas can be used as a tool for mitigating the loss of phylogenetic diversity in avian communities^39^ and delivering environmental ecosystem services.^70^ Furthermore, when evaluating the effects of urbanization on the overall biodiversity, it is important to consider the effect of different types of urban areas within the cities (e.g., based on the building density and spatial arrangement of green areas). A negative association between the overall evolutionary distinctiveness of bird assemblages and the density of human settlements was consistent even when considering the potential confounding effect of species richness. Thus, our results highlighted the fact that an urbanization gradient could modify not only the total number of species and the functional diversity of bird communities^71^^,^^72^ but also their evolutionary distinctiveness, increasing the concern raised about the overall negative effect of the expansion of urban areas on biodiversity.^73^^,^^74^

### Limitations of the study

Our findings have relevant implications for conservation planning within cities. However, some potential limitations of the research should be noted. Our study was conducted only during the breeding season, so we have no information regarding the effects of urbanization on avian communities wintering in urban areas. Additionally, in our models, no natural sites (areas without urbanization) were included and, with a high probability, the contrast between urban bird communities and assemblages from natural environments should be even sharper.^23^ In our study, instead, we preferred to focus on the differences in evolutionary distinctiveness across a gradient of urbanization. Finally, the inclusion of nocturnal species (excluded from our analysis) could highlight such potential differences more markedly.

### Conclusion

In conclusion, we suggest that metrics related to the evolutionary uniqueness of species assemblages have to play a role when assessing the effects of urbanization on the biotic homogenization of urban wildlife because they can be used to identify local conservation priorities^75^ or measure the overall impact of highly densely building areas on the avian communities inhabiting cities.^24^ The enrichment of bird communities characterized by a large amount of phylogenetic diversity should be a common target for the conservation of urban biodiversity. Afterward, urban planning must work in synergy with conservation biology and ecological studies to better understand the mechanisms that attract more unique species within the cities, potentially increasing communities’ resilience. In this way, a multidisciplinary aim of harmonization between urban settlement development and the protection of biodiversity^76^ can be more effectively achieved.

## STAR★Methods

### Key resources table

**Table:** 

REAGENT or RESOURCE	SOURCE	IDENTIFIER
**Data**		

Community evolutionary distinctiveness of bird assemblages in a gradient of urbanization.	This paper	https://figshare.com/articles/dataset/Community_evolutionary_distinctiveness_of_bird_assemblages_in_a_gradient_of_urbanization/24886929
Evolutionary distinctiveness score for bird species of the World	www.edgeofexistence.org	N/A

**Software**		

R software version 4.1.1	R Development Core Team^60^	https://www.r-project.org/

### Resource availability

#### Lead contact

Further information and requests for resources and reagents should be directed to and will be fulfilled by the lead contact, Federico Morelli (fmorellius@gmail.com).

#### Materials availability

Data used in this study have been made available in the Supplementary Material. Evolutionary Distinctiveness (ED) scores for all bird species recorded in our study were freely downloaded from www.edgeofexistence.org and are based on the bird phylogeny published by Jetz et al. 2014.

#### Data and code availability

All original data has been deposited at Figshare and is publicly available as of the date of publication. https://doi.org/10.6084/m9.figshare.24886929. Any additional information or codes required to re-analyze the data reported in this paper is available from the lead contact upon request.

### Experimental model and study participant details

Expert ornithologists collected data on bird species composition through standardised point counts during the 2018 breeding season in seventeen cities located along a continent-wide latitudinal gradient, encompassing ten European countries from the Mediterranean area up to the Artic Circle. Point counts were performed in a gradient of urbanization within each European city. Full details regarding the field work are available in the following publication: Morelli, F., Benedetti, Y., Ibáñez-Álamo, J.D., Tryjanowski, P., Jokimäki, J., Kaisanlahti-jokimäki, M., Suhonen, J., Díaz, M., Møller, A.P., Moravec, D. et al. (2021). Effects of urbanization on taxonomic, functional and phylogenetic avian diversity in Europe. Sci. Total Environ. 795, 148874. 10.1016/j.scitotenv.2021.148874.

### Method details

Calculating the community evolutionary distinctiveness (CED) - Using the ED score for each bird species present in a community, we estimated the CED as the average ED, considering all species belonging to the community. The CED of a community is calculated as follows:

$$\text{CED}=\sum_{i=1}^{n}\frac{{ED}_{i}}{N_{tot}}$$

Where ED_*i*_ is the evolutionary distinctiveness score for each species *i*, divided by the number of all species recorded in the community, N_tot_.

CED was then calculated for each point count, in each city, in the gradient of urbanization (low, medium or high building density).

### Quantification and statistical analysis

#### Modeling procedure

A generalized linear mixed effects regression model (GLMM) was run, using CED as response variable after a log-transformation, while the predictor variables were bird species richness, latitude, and level of urbanization (low, medium, high). “City” was included as a random effect in the model to account for possible differences among cities and alleviate any potential spatial autocorrelation. The model was fitted using the package ‘lme4’ for R,^54^ while the model output was obtained using the package ‘jtools’ for R.^55^ Potential multicollinearity among predictors was assessed using the variance inflation factor (VIF)^56^ in the ‘car’ package for R.^57^ Only predictors with VIF<1.5 were incorporated into the model procedure.^58^

#### Validation

To assess the validity and robustness of the results, the goodness of fit of the model was evaluated using conditional and marginal coefficient of determination for GLMM, with the function ‘r.squaredGLMM’ from the ‘MuMIn’ package for R.^59^

## Supplemental information

Document S1. Figures S1–S3 and Table S2

Table S1. A list of 134 bird species recorded at 1618 point counts distributed in 17 European cities focused on this study, with the evolutionary distinctiveness (ED) score, total number of observations (N) and total frequency of occurrence regarding the total number of point counts (%), for each category of urban based on the building density (low, medium, high) The order of species in the table is based on their relative abundance. The ED score was extracted at www.edgeofexistence.org.
