# Supplementary material for: Dense city centers support less evolutionary unique bird communities than sparser urban areas
Source: iScience. 2024 Jan 18;27(2):108945. doi: 10.1016/j.isci.2024.108945 (PMC10844830; doi:10.1016/j.isci.2024.108945)

iScience, Volume *27*

Supplemental information

Dense city centers support less evolutionary unique bird communities than sparser urban areas

Federico Morelli, Jiri Reif, Mario Díaz, Piotr Tryjanowski, Juan Diego Ibáñez-Álamo, Jukka Suhonen, Jukka Jokimäki, Marja-Liisa Kaisanlahti-Jokimäki, Anders Pape Møller, Leszek Jerzak, Raphaël Bussière, Marko Mägi, Theodoros Kominos, Antonia Galanaki, Nikos Bukas, Gábor Markó, Fabio Pruscini, Olaf Ciebiera, and Yanina Benedetti

Electronic Supplementary Material - "Dense city centres support less evolutionary unique bird communities than sparser urban areas"

Table S2. Variables which were used to investigate the effects of building density (level of urban, low, medium and high) in 17 European cities on the community evolutionary distinctiveness and the Variance Inflation Factor (VIF).

**Variable**	**VIF**	**Df**
Bird species richness	1.271	1
Level urban	1.187	2
Latitude	1.087	1

Figure S1. A gradient of cover for buildings or built up (A), grass (B), bush (C) and trees (D) in each type of area within the city, classified following the level of urban (low, medium and high), represented by a colourimetric map. Grey colour (e.g., Orsay for bush and tree cover) indicates missing data.

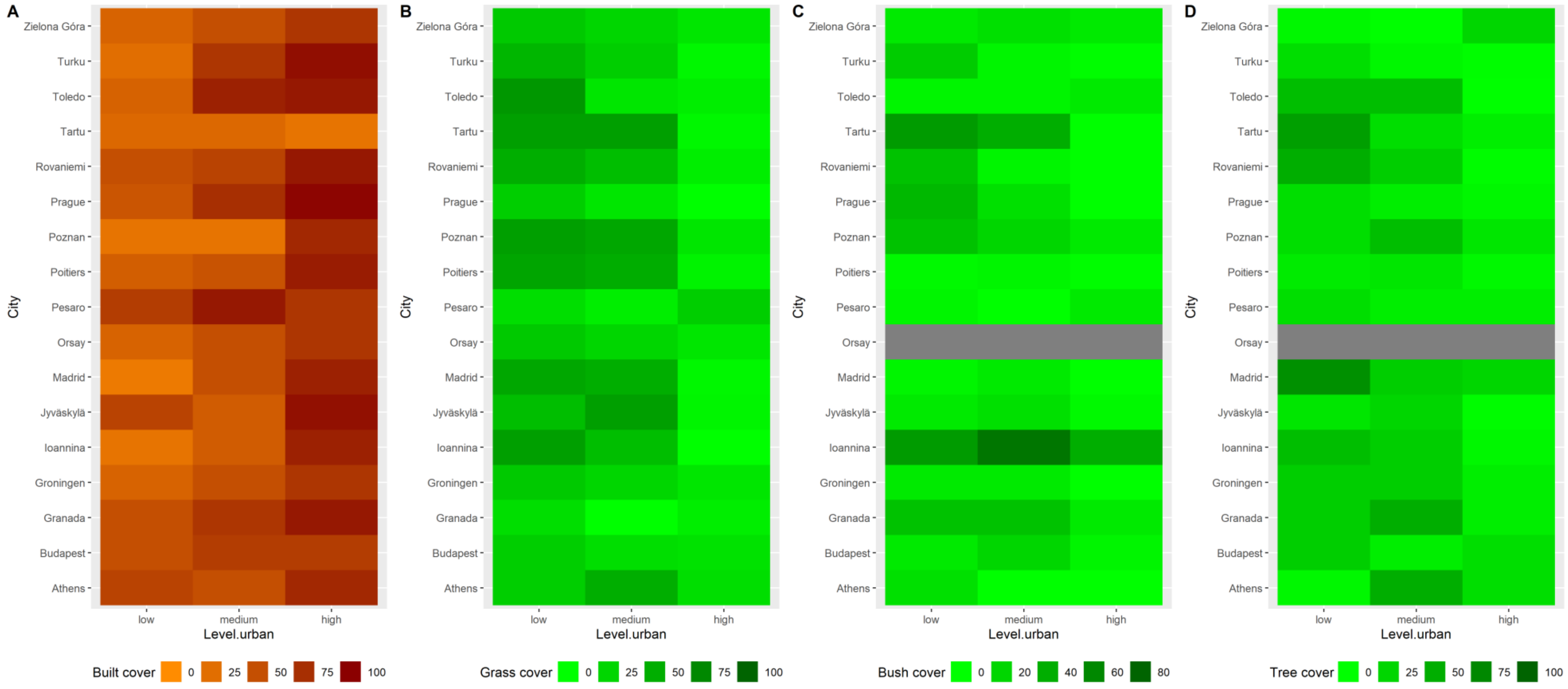

Figure S2. Diagnostic plots for the variable community evolutionary distinctiveness (CED), which was used as the response variable in the modelling procedure, after normalization by a log transformation.

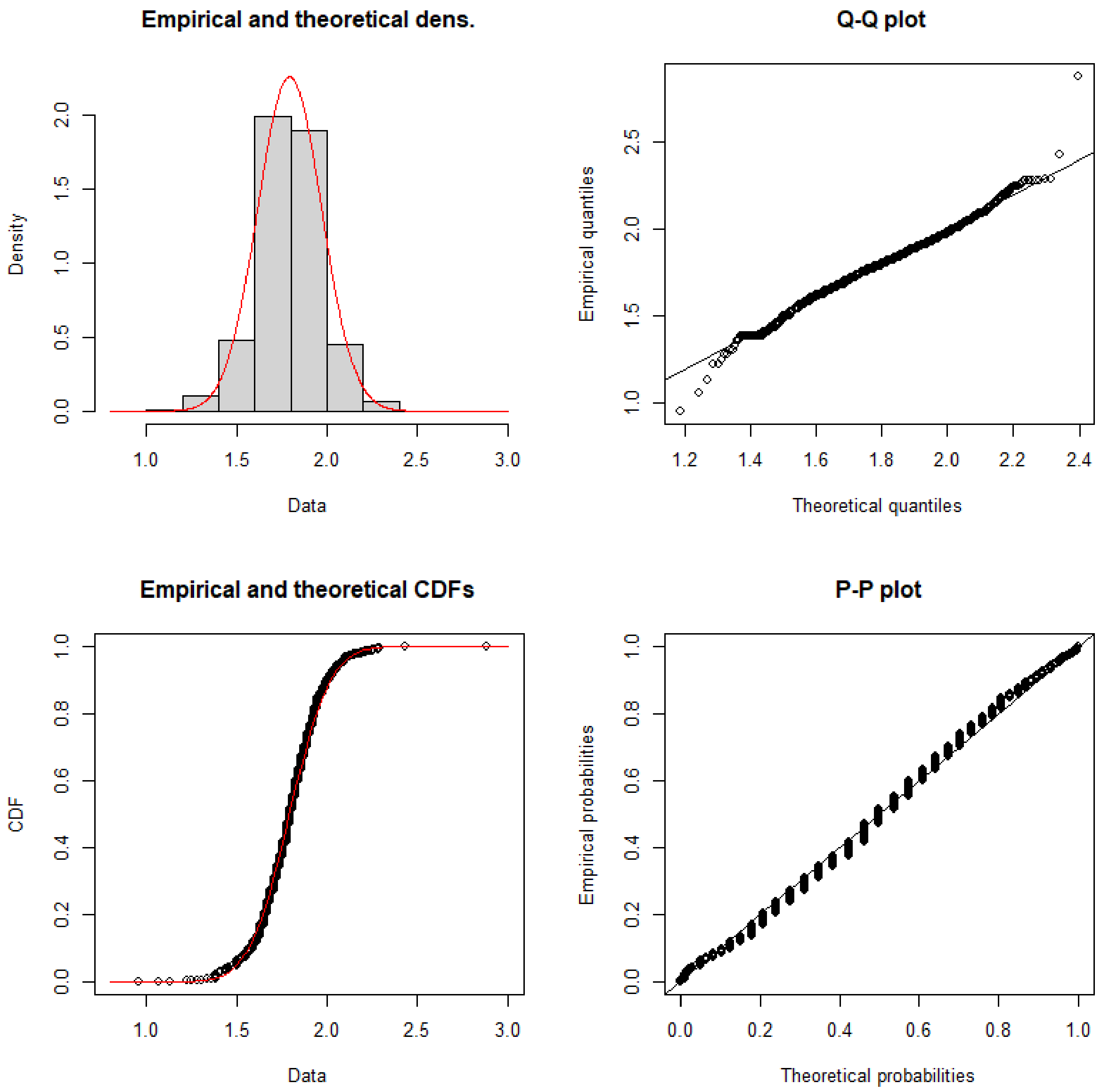

Figure S3. Maximum values of evolutionary distinctiveness scores of bird species in a gradient of urbanization (low, medium and high) within seventeen European cities. The raincloud plot shows the raw data, probability density and summary statistics such as the median (black bar in the middle of the coloured rectangles), mean (grey circles), upper and lower quartiles by presenting individual data, a violin plot and a boxplot together.

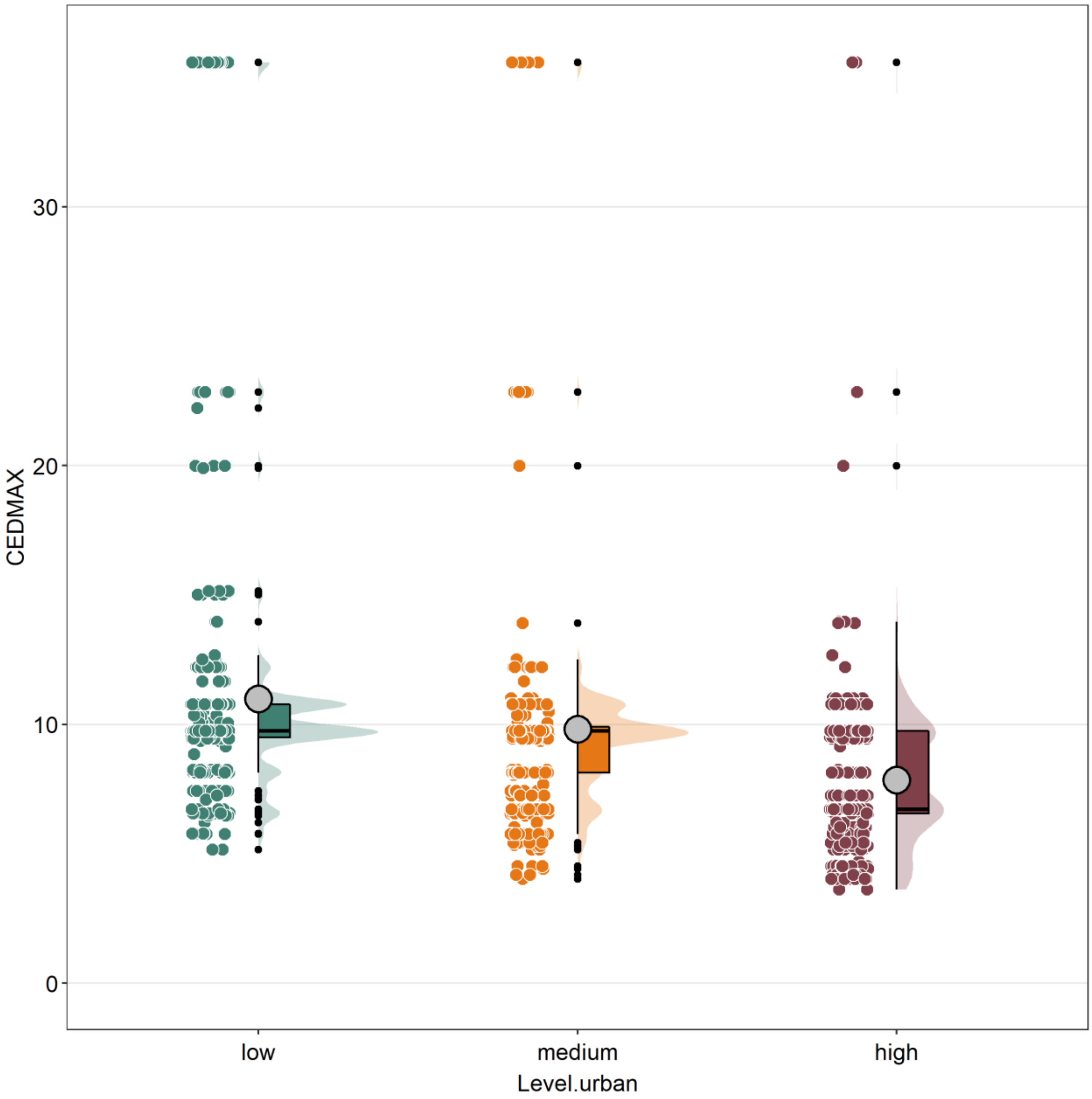

Supplement: Document S1. Figures S1–S3 and Table S2 [file mmc1.pdf]
